# Impact of Gamma rays and DBD plasma treatments on wastewater treatment

**DOI:** 10.1038/s41598-018-21001-z

**Published:** 2018-02-13

**Authors:** Pankaj Attri, Fumiyoshi Tochikubo, Ji Hoon Park, Eun Ha Choi, Kazunori Koga, Masaharu Shiratani

**Affiliations:** 1 Plasma Bioscience Research Center/Department of Electrical and Biological Physics, Kwangwoon University, Seoul, 01897 Korea; 2 Faculty of Information Science and Electrical Engineering, Kyushu University, Fukuoka, Japan; 3 Department of Electrical and Electronic Engineering, Tokyo Metropolitan University, Tokyo, Japan

## Abstract

The rapid growth in world population brings with it the need for improvement in the current technology for water purification, in order to provide adequate potable water to everyone. Although an advanced oxidation process has been used to purify wastewater, its action mechanism is still not clear. Therefore, in the present study we treat dye-polluted water with gamma rays and dielectric barrier discharge (DBD) plasma. We study the wastewater treatment efficiency of gamma rays and DBD plasma at different absorbed doses, and at different time intervals, respectively. Methyl orange and methylene blue dyes are taken as model dyes. To understand the effects of environment and humidity on the decolorization of these dyes, we use various gas mixtures in the DBD plasma reactor. In the plasma reactor, we use the ambient air and ambient air + other gas (oxygen, nitrogen, and argon) mixtures, respectively, for the treatment of dyes. Additionally, we study the humidity effect on the decolorization of dyes with air plasma. Moreover, we also perform plasma simulation in different environment conditions, to understand which major radicals are generated during the plasma treatments, and determine their probable densities.

## Introduction

In recent years, drastic increases in pollutants in water resources have been detected^1–3^. The presence of organic pollutants has given rise to serious concerns for aquatic life and public health. Among all organic pollutants, dye wastewater is of main concern, and there has been rapid increase in dye wastewater that comes mainly from the dying textile, ink production, chemical analysis, and ceramics industries. There are more than 10,000 different types of pigment and dyes used by industries, and more than 0.7 million tons of dyes are synthesized annually worldwide^4^. Additionally, 15% dyes that are lost during dye synthesis and processing end up in wastewater^5^. The decolorization of wastewater in textile industries can help in the reuse of this water for further textile processing^6,7^. This can also help in taking precautions against the diseases caused by these dyes, because most synthetic dyes are carcinogenic and toxic in nature. When discharged into the environment, the organic dyes are toxic for both flora and fauna, because organic dyes absorb and reflect sunlight entering the water, which results in the killing of aquatic species, as well as the bacteria that are used to degrade impurities in the water^8,9^. But decolorization is not an easy task, because dye consists of very stable molecular structures. Therefore, many conventional methods are not useful, or they have very low efficiency for the decolorization of dye. Hence, to solve this problem, many techniques are being developed based on advanced oxidation processes (AOPs)^10^.

Of all the AOPs, cold atmospheric plasma (CAP) is nowadays studied frequently for wastewater treatment^11–16^, because it has the ability to achieve enhanced gas phase and liquid phase chemistry at relatively low gas temperature with low power consumption. Additionally, this technology is also widely used for various other applications, such as surface modification, material processing and synthesis, and biomedical, among many others^17–29^. There are different types of CAP devices, but the DBD reactor is the most commonly used CAP device to degrade organic pollutants, AR88 acid, azo dye Orange II, methylene blue dye, and methyl orange dye^11,12,30–33^. Moreover, many research groups have used methylene blue and methyl orange as model dyes to analysis the efficiency of their plasma devices^34–39^. Liu *et al*. decolorized methyl orange to 93% after 15 min treatment with glow discharge plasma^34^. The combination of Ag/TiO_2_ nanocomposite with APPJ was used to degrade methyl orange^35^. Another research group showed that APPJ reactor could enhance the catalytic activity of Co_3_O_4_ for methyl orange degradation^36^. Further, Grabowski *et al*. showed that pulsed corona discharge could break down methylene blue and methyl orange dyes^37^. Also, ≈99% methyl orange degradation was studied using pulsed high voltage discharge plasma, when discharge plasma was 20,000×^38^.

The other AOP is gamma irradiation, which was also studied for the treatment of wastewater^40–44^. Previous study used gamma irradiation in the presence of H_2_O_2_ to decontaminate and decolorize textile wastewater; they found that the addition of H_2_O_2_ could help promote the degradation of organic materials^40^. In another study, Reactive Red 120 dye was degraded using gamma irradiation (5.94 kGy), and degradation was influenced by the change in pH^41^. Additionally, it was reported that combined γ-rays and FeSO_4_ could effectively degrade sulfadiazine (SD) solution samples^42^. It was also reported that the degradation of methyl orange using γ-rays increases as the dose rate increases^43^. Recent study has reported that gamma radiation could successfully degrade textile wastewater that contains coloring materials and organic pollutants^44^. Thus, in the present work, we study the efficiency of gamma rays and dielectric barrier discharge (DBD) plasma on the decolorization of dyes as a function of time. We use methyl orange and methylene blue as the model dyes, and different feeding gases (O_2_, N_2_, Ar and Air) in the DBD reactor to analyze the effect of gases on the decolorization of both dyes. Further, we analyze the reactive species (RS) in DI water after the gamma ray and DBD plasma treatments (with all feeding gases) for different dose rates ((228, 424.4, and 1,136) Gy/h), and for different time intervals ((10 and 20) min), respectively. We also simulate the RS densities in various feeding gas mixtures, to understand the mechanism of decolorization of dyes by DBD plasma.

## Experimental Section

### Dielectric barrier discharge

**Figure 1:**
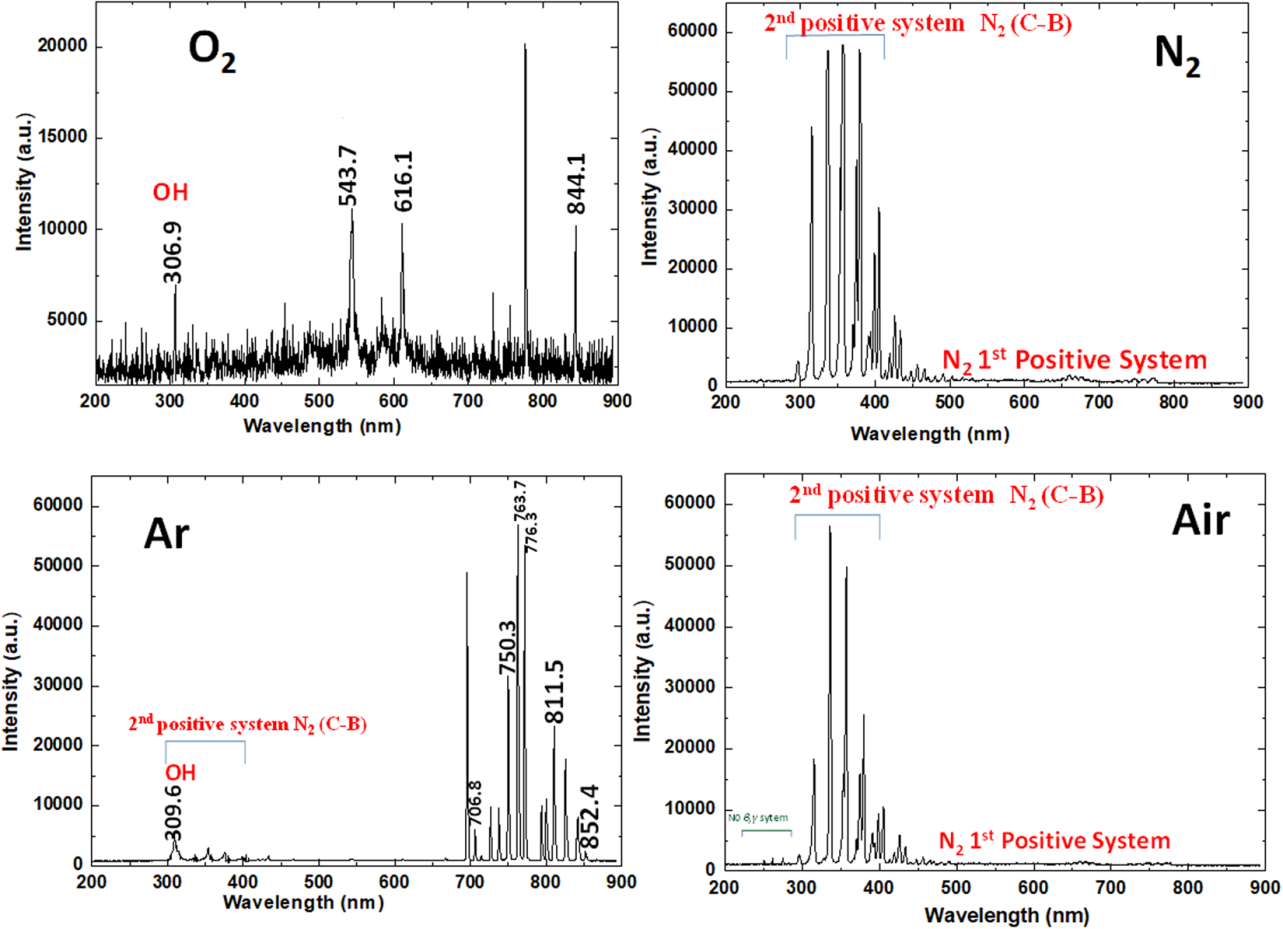
Optical emission spectra of O_2_, N_2_, Ar, and Air feeding gases plasma.

Experiments were carried out using a DBD device, which was set in a chamber equipped with a rotary vacuum pump and a gas cylinder. The gases used in the chamber were O_2_, N_2_, Ar, and Air. The DBD device consisted of stainless rod electrodes of 1 mm in outer diameter and 60 mm in length, covered with a ceramic tube of 2 mm in outer diameter. The electrodes were arranged in parallel, with a spacing of 0.2 mm. The discharge area of the DBD was 3.12 cm^2^, the power supply provided was LHV-10AC, with slidac voltage of 100 V. The discharge were generated between two electrode and number of gap is 39. A capacitor (100 pF) was connected between the power supply and electrode. The discharge voltage and current were measured by high-voltage probe (Tektronix, P6015A) and Rogowski coil (URD, CTL-28-S90-05Z-1R1), respectively. The corresponding discharge power density was deduced from a voltage/charge Lissajous plot in the presence of each gas, as described below. The OES spectra of the DBD emission in the presence of all gases were recorded by the use of HR4000CG-UV-NIR (Ocean Optics, FL, USA). The signal was accumulated for 5 min, and the data were analyzed using the Origin 8.0 software package. Figure 1 shows the emission spectra that were recorded.

### Materials and analysis

Methyl orange and methylene blue were purchased from Sigma Aldrich Pvt. Ltd. The stock solutions of methyl orange and methylene blue were prepared by dissolving the required amount of analytical grade dye in Millipore water. The experimental solutions (200 mg/L) were obtained by diluting the stock solution in accurate proportions. Degradation of dye was monitored by UV-Vis Spectrophotometer S-3100, of wavelength resolution 0.95 nm, wavelength accuracy ±0.5 nm, and wavelength reproducibility of ±0.02 nm. Absorption for methyl orange (MO) and methylene blue (MB) was measured at (505 and 665) nm, respectively. The degradation percentage and efficiency (g/kWh) were calculated following a reported method^33^. The H_2_O_2_ was measured using titanyl ion^19,20,24^, and NO was detected using 4-amino-5-methylamino-2′,7′-difluorofluorescein (DAF-FM)^21,24,26^. OH was measured using terephthalic acid (20 mM), as per the procedure given in earlier research work^45^. NO_2_^−^/NO_3_^−^ was measured using the Nitrite/Nitrate Assay Kit, colorimetric supplied by Aldrich Chemical Co. (USA). Meanwhile, the NO_3_^−^ concentration was cross-checked using the Acorn Series ION 6 Meter (pH/mV/°C meter), nitrate electrode, from Oakton Instruments, USA^45,46^.

### Sample preparation and treatment condition

The 200 mg/mL concentrations of both dyes were prepared from stock solution, as mentioned above. For the gamma rays treatment, we added the 20 mL of methyl orange or methylene blue dye in quartz petri dish, and placed in front of the Cobalt-60 gamma-ray Irradiator at different positions, where the different dose rates (Gy/h) were given to the dyes (the positions of different doses were standardized by the Center for Accelerator and Beam Applied Science, Kyushu University, Japan).

For the plasma treatment, we similarly used 20 mL of the methyl orange or methylene blue dye in quartz petri dish, and placed below the DBD source at a distance of 4 mm, inside the chamber, and then closed the chamber. First, we created a vacuum of  apporixamtely 10 Pa using rotary pump (at higher than this vacuum, the dye solution started coming out of the petri dish; therefore, we fixed this condition for our treatment), and then inserted the gas (different gases as per required for the experiment) to make the final pressure equal to atmospheric pressure the addition of gases to the chamber makes final pressure equal to atmospheric pressure, assume that there is air ≈40% (because vacuum created ≈60%) and specific gas added to ≈60%. Through this we assume that the air ≈40% and specific gas ≈60% present in the treatment chamber.

### Simulation details

The composition of molecules in the DBD plasma has been calculated using the zero-dimensional rate equations of chemical reactions^47^. In the real situation, the molecular density profiles strongly depend on the position. There are small filament-like discharges in the plasmas. The molecular density in the discharges is high. Since numerous numbers of discharges are randomly generated in the discharge space, we assumed that the densities of species, including electrons, are uniformly distributed in the plasma, and that the electron energy distribution is the Maxwell-Boltzmann distribution. To calculate their density, we adopted a set of rate equations that are given by:

1 $$\frac{\partial {n}_{i}}{\partial t}={\sum }_{i}{k}_{ij}{n}_{i}{n}_{j}-{\sum }_{l,m}{k}_{lm}{n}_{l}{n}_{m}$$

where, n is the density of the molecules, and k is the reaction rate coefficient. Subscripts *i*, *j*, *l*, and *m* show species that are considered in the chemical reaction. In this work, we simply considered the radical production by electron impact dissociation of the parent gas at electron temperature of 1 eV and electron density of 5 × 10^15^ cm^−3^, and the reactions between neutral species including radicals, until the calculated results reach the steady state.

## Results and Discussion

### DBD plasma parameters and analysis of reactive species generated in the gas phase

**Figure 2:**
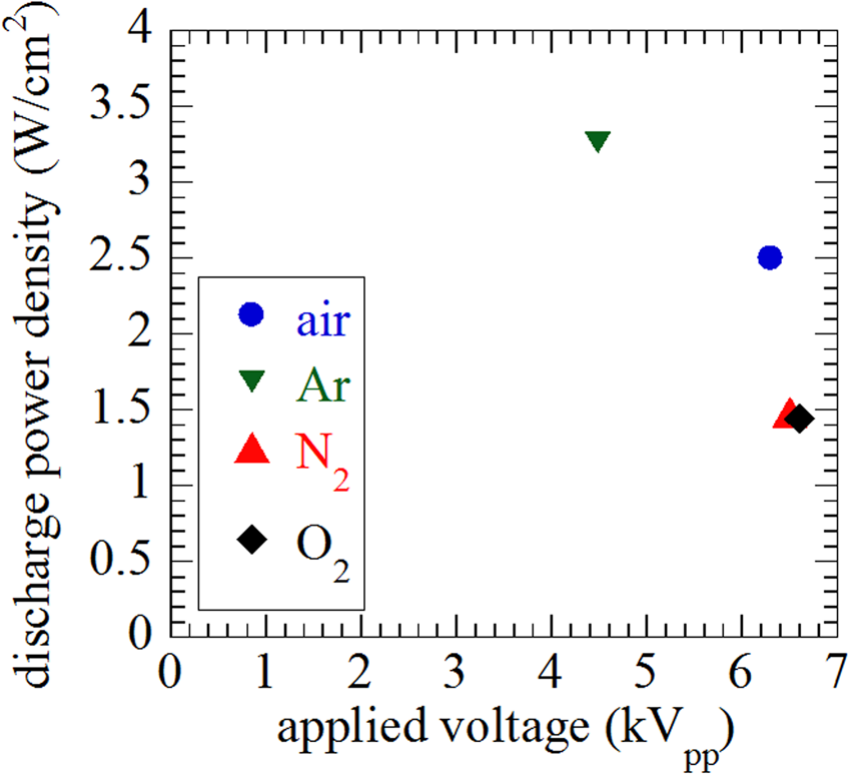
The DBD plasma parameters using different feeding gases.

In this work, we used DBD plasma that was generated in O_2_, N_2_, Ar, and Air gaseous environment. Figure 2 shows the dependence of power density as a function of voltage that we obtained, using these gases in the treatment chamber at atmospheric pressure. Table 1 shows that for the O_2_ environment, the discharge power density was 1.44 W.cm^−2^ and the discharge power was 4.5 W, with a discharge frequency of 10.6 kHz. For the N_2_ gas plasma generated, the discharge power density was 1.48 W.cm^−2^, and the discharge power was 4.6 W, with a discharge frequency of 10.6 kHz. For the Ar gas plasma, the discharge power density was 3.26 W.cm^−2^, and the discharge power was 10.2 W, with a discharge frequency of 10.6 kHz. For only Air plasma, the discharge power density was 2.5 W.cm^−2^, and the discharge power was 7.8 W, with a discharge frequency of 10.7 kHz. Hence, the N_2_ and O_2_ gas plasma have similar discharge power, but in our system, the discharge power of pure air plasma is quite high. The discharge voltage has been changed during different gases plasma because the ionization energy or electron impact ionization cross section is different among the gases. And in current work, the input voltage to pulse power source was set to be same value for the gases discharge, which results in different discharge power.

**Table 1:** DBD plasma parameters in the presence of different feeding gases.

Feeding Gases	Applied voltage [kV_pp_]	Discharge power density [W/cm^2^]	Discharge power [W]	Discharge frequency [kHz]
O_2_	6.60	1.44	4.497	10.6
N_2_	6.50	1.48	4.611	10.6
Ar	4.48	3.26	10.181	10.6
Air	6.30	2.50	7.796	10.7

Figure 1 shows the RONS generated in gaseous phase for different combinations of gases, using optical emission spectroscopy (OES). During the O_2_-DBD plasma, we observed a high-intensity ^•^OH peak at 306.9 nm and O_2_ 1^st^ positive system at (777.2 and 844.1) nm, respectively. For N_2_-DBD plasma, we detected the ^•^OH peak at 306.9 nm, N_2_ second-positive system (C^3^П_u_–B^3^П_g_) peaks at (294.5, 314.1, 335.3, 353.7, and 379.0) nm, and also observed the N_2_ first-positive system (B^3^П_g_–A^3^П_u_^+^) peaks in the range (500–700) nm. Moreover, for Ar-DBD plasma, we observed a small ^•^OH peak at 309.6 nm, and additional Ar lines in the range (600–800) nm. For the Air plasma, we observed small emission lines in the range (200–250) nm that belong to the molecular NO β, γ system. Strong peaks were detected for the N_2_ second-positive system (C^3^П_u_–B^3^П_g_) at (294.5, 314.1, 335.3, 353.7, and 379.0) nm, and weak peaks were detected for the N_2_ first positive system (B^3^П_g_–A^3^П_u_^+^).

### Analysis of reactive oxygen and nitrogen species and pH generated in DI water after the treatment with gamma rays and DBD plasma in different gaseous environment

**Figure 3:**
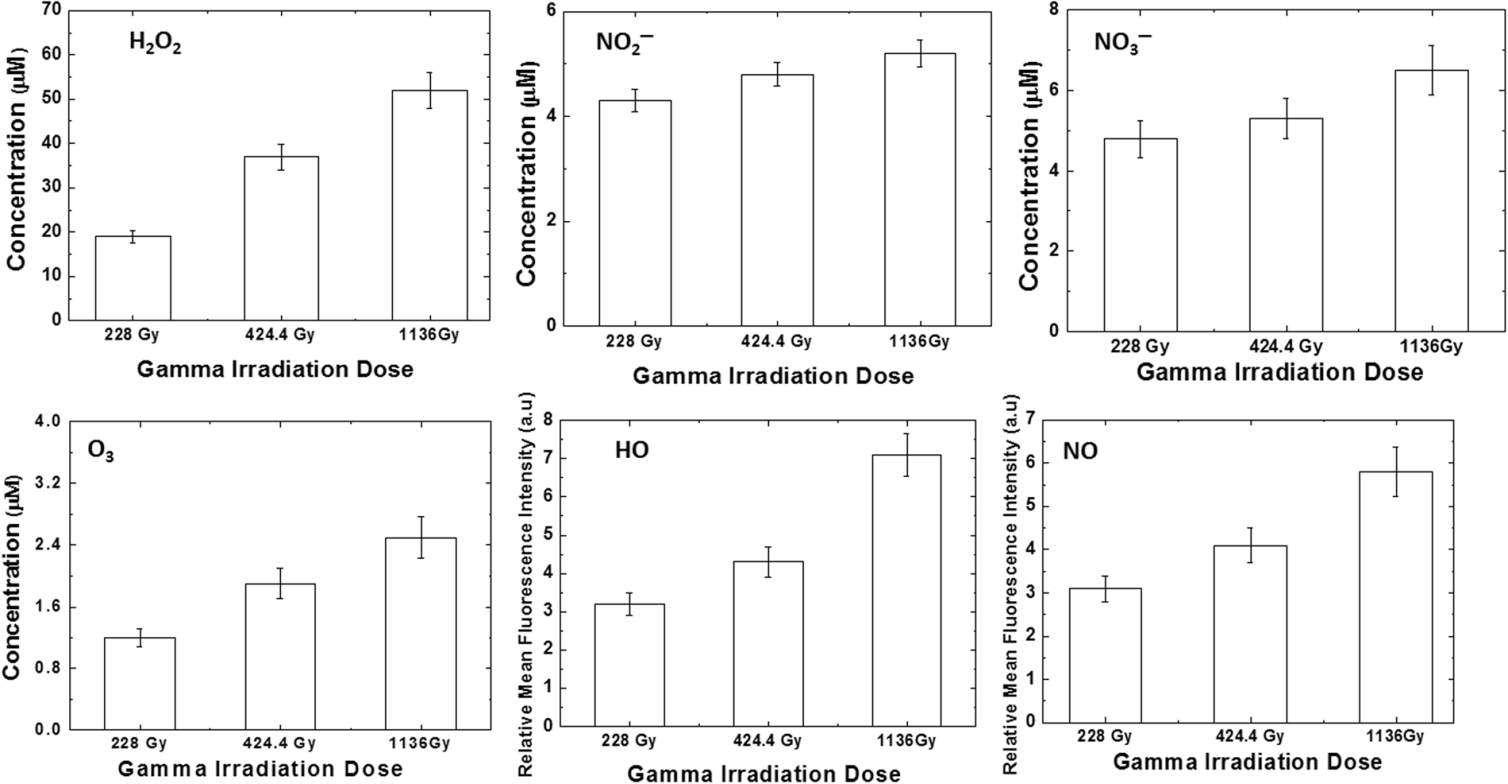
Analysis of reactive oxygen and nitrogen species detection in water after treatment with different gamma rays absorbed doses of (228, 424 and 1,136) Gy.

**Figure 4:**
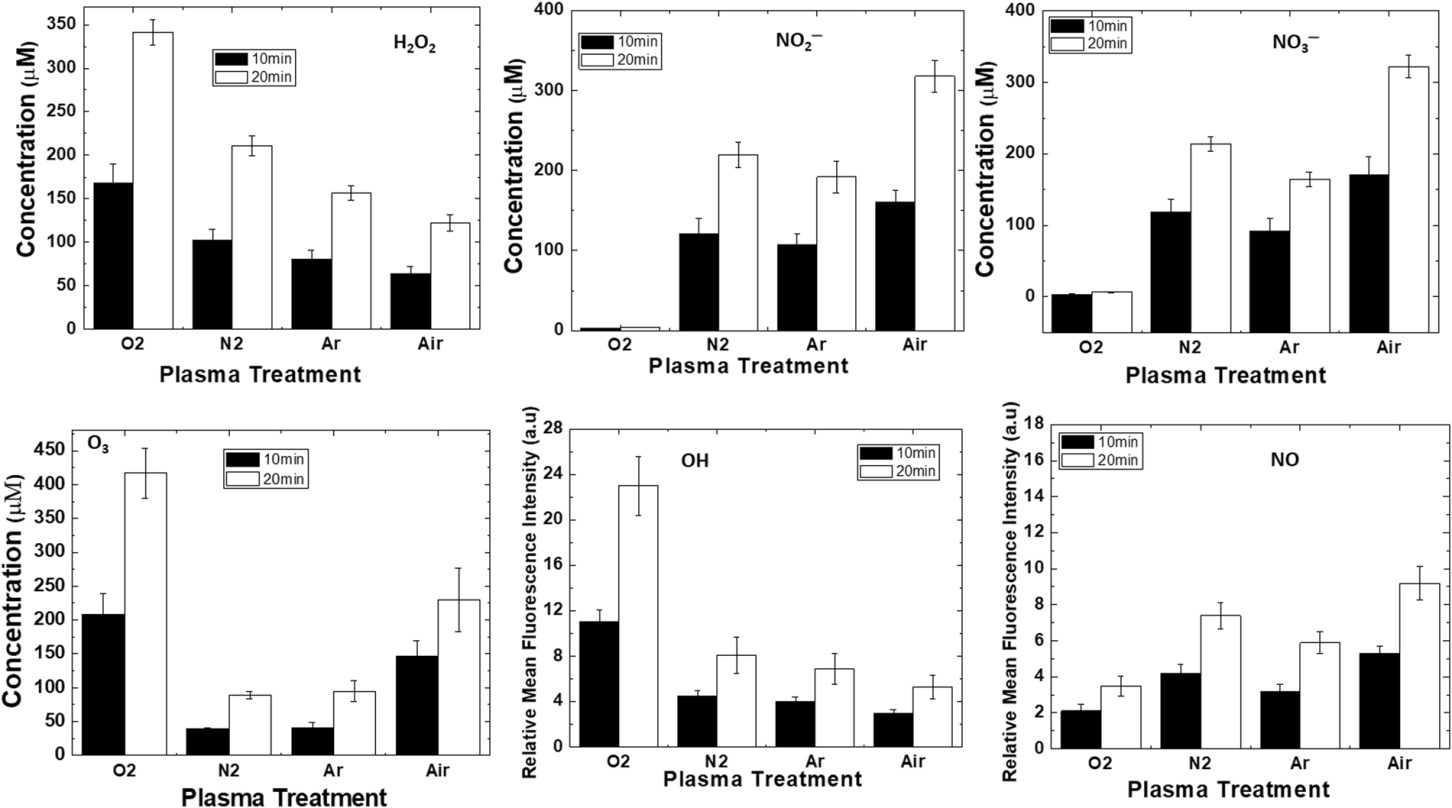
Analysis of reactive oxygen and nitrogen species detection in water after treatment with DBD plasma using different feeding gases (O_2_, N_2_, Ar, and Air) for different time intervals of (10 and 20) min.

Plasma irradiation consists of RS that are either short-lived or long-lived, and the concentrations of these RS mainly depend upon the electron temperature, electron density, feeding gases, and environmental conditions. Figures 3 and 4 show the concentration of RS using chemical analysis after the treatment with gamma rays and DBD plasma on DI water. We analyzed the formation of RONS, such as hydrogen peroxide (H_2_O_2_), nitrites (NO_2_^−^), nitrates (NO_3_^−^), ozone (O_3_), hydroxyl radicals (^•^OH), and nitric oxide (NO^•^). After the gamma ray treatment on the DI water for (228, 424.4, and 1,136) Gy absorbed dose, the H_2_O_2_ concentration was (19, 37, and 52) μM, respectively (Fig. 3); however, under the same conditions, the NO_2_^−^ concentration was ≈(4.3, 4.8, and 5.2) μM, respectively, while the NO_3_^−^ concentration was ≈(4.8, 5.3, and 6.5) μM, respectively; additionally, the O_3_ concentration was (1.2, 1.9, and 2.5) μM, respectively (Fig. 3). Further, we have detected the ^•^OH and NO^•^ presence in the DI water after different absorbed dose of gamma rays of (228, 424.4, and 1,136) Gy (Fig. 3). We observed that the ^•^OH and NO^•^ fluorescence intensity was least for the 228 Gy, and highest for the 1,136 Gy absorbed dose. These results show that as the treatment time of gamma rays increases, the fluorescence intensity also increases, which resembles the concentration of ^•^OH and NO^•^ increases.

Figure 4 shows that in the plasma reactor, we used the ambient Air and Air (40%)+ other gases (60%) (oxygen, nitrogen, and argon) mixture conditions, respectively, with humidity of 40% and treatment time of (10 and 20 min). In these conditions, we analyzed the concentration of H_2_O_2_, NO_2_^−^, NO_3_^−^, O_3_, ^•^OH, and NO^•^ after the DBD plasma. After 10 min DBD plasma treatment in the presence of Air + O_2_, Air + N_2_, Air + Ar, and Air, the H_2_O_2_ concentration was ≈(168, 102, 81, and 64) μM, respectively (Fig. 4); however after the 20 min treatment in the same condition, the H_2_O_2_ concentration was ≈(341, 211, 157, and 122) μM, respectively. On the other hand, after 10 min DBD plasma in the presence of Air + O_2_, Air + N_2_, Air + Ar and Air, the NO_2_^−^ and NO_3_^−^ concentrations were ≈(3.1, 121, 108, and 161) μM, and (3.6, 119, 92, and 171) μM, respectively (Fig. 4); whereas, after 20 min DBD plasma under the same conditions, the NO_2_^−^ and NO_3_^−^ concentrations were ≈(4.4, 220, 192, and 318) μM, and (6.4, 214, 164, and 322) μM, respectively (Fig. 4). For the 10 min treatment of DBD plasma, the concentration of O_3_ also varied from gas to gas (O_2_, N_2_, Ar, and Air) in the DBD reactor, such as ≈(208, 39, 41, and 147) μM, respectively; meanwhile, after the 20 min treatment, the O_3_ concentration for the O_2_, N_2_, Ar, and Air DBD plasma was ≈(417, 89, 95, and 230) μM (Fig. 4). Additionally, we detected the ^•^OH and NO^•^ in the DI water after the treatment of DBD plasma in the presence of Air + O_2_, Air + N_2_, Air + Ar, and Air for (10 and 20) min (Fig. 4); we observed that for both treatment times (10 and 20 min), the ^•^OH fluorescence intensity was least for the Air plasma, and highest for the Air + O_2_ plasma. As the treatment time increased, the fluorescence intensity also increased, which resembled the ^•^OH concentration increases. Whereas, Air + N_2_ DBD plasma showed higher fluorescence intensity than the Air + Ar DBD plasma, but the difference between the two was not significant for all treatment times. Conversely, the NO^•^ fluorescence intensity was least for the Air + O_2_ plasma, and highest for the Air plasma. The fluorescence intensity that resembled the concentration of NO^•^ increased as the treatment time increased. Thus, the concentration of RONS depends upon the types of gas used in the chamber and the treatment time; as the treatment time increases, the RONS concentration increases.

Further, we have checked the change in pH of DI water after the treatment with gamma rays and DBD plasma, as shown in Fig. S1. Figure S1(a) shows the slight decrease in pH of water after the treatment gamma rays. As the gamma dose rate increases, the pH of water slightly decreases. Moreover among all the studied gases plasma, Fig. S1b shows that the pH of water decreases most for the air plasma treatment, and least for the O_2_ plasma treatment. After 10 min treatment, the pH decreases to 2.8 for air plasma, while decreasing to 4.9 for O_2_ plasma.

### Dyes decolorization efficiencies of gamma rays and DBD plasma

**Figure 5:**
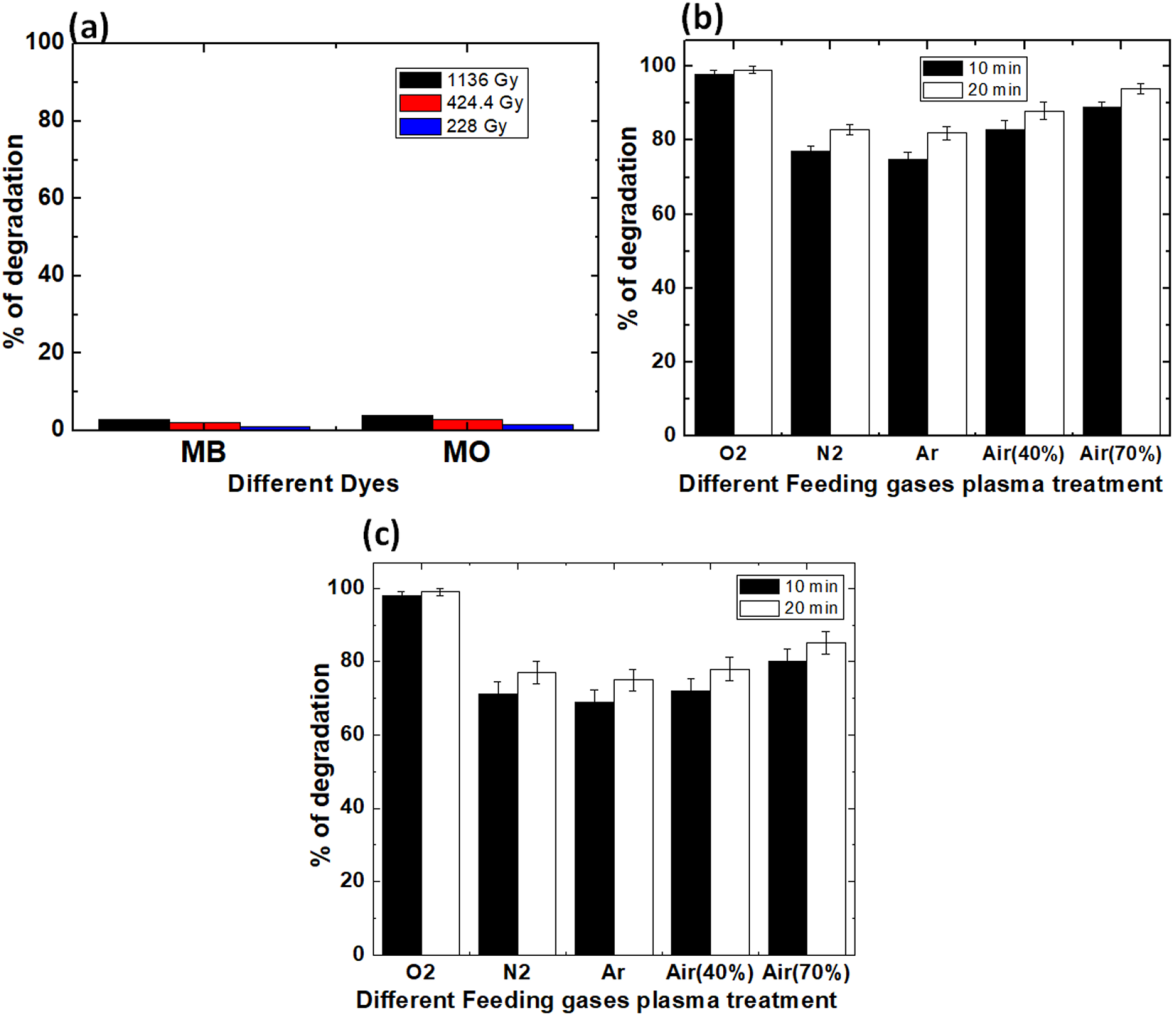
Decolorization percentages obtained by UV-Vis spectroscopy after (**a**) gamma treatments for MB and MO; (**b**) MO with DBD plasma treatment and (**c**) MB with DBD plasma treatment.

The degradation of organic dyes (methyl orange and methylene blue) using gamma rays and DBD plasma in the presence of different gases (O_2_, N_2_, Ar, and Air) is a complex process. The gamma rays and DBD plasma generate RS in the gas phase or gas-liquid interfaces that transport to the liquid layer, and help in generating secondary radicals, due to the high reactive potential of the primary RS. These primary and secondary RS help break down the dye molecules into intermediate products or final stable products. In the present experiment, we did not employ any forced stirring; hence the mixing in the system is limited to diffusion only. Figure 5 analyzes the decolorization of methyl orange and methylene blue dyes after treatment with different dose of gamma rays of (228, 424.4, and 1,136) Gy. Figure 5(a) shows that after exposure with gamma rays for (228, 424.4, and 1,136) Gy, the degradation for methyl orange dye was (2, 3, and 4) %, respectively; on the other hand, the degradation for methylene blue was (1, 2, and 3) %, respectively.

Figure 5(b) shows the treatment of methyl orange with DBD plasma in the presence of Air + O_2_, Air + N_2_, Air + Ar, and Air, with humidity of 40%. After 10 min treatment, the decolorization was (98, 77, 75, and 83) %, respectively; while after 20 min treatment, the decolorization was (99, 83, 82, and 88) %, respectively. Figure 5(c) shows the treatment of methylene blue dye with DBD plasma in the presence of Air + O_2_, Air + N_2_, Air + Ar, and Air. After 10 min treatment, the decolorization was (98, 71, 69, and 72) %, respectively; while after 20 min treatment, the decolorization was (99, 75, 73, and 78) %, respectively. Additionally, for the treatment of methyl orange with Air DBD plasma having humidity of 70%, the decolorization after (10 and 20) min treatment was (89 and 94) %, respectively; whereas, for treatment of methylene blue dye, the decolorization after (10 and 20) min treatment was (80 and 85) %, respectively.

### Plasma efficiency in the presence of different gases

This study reveals that in the presence of different feeding gases, DBD plasma source does not degrade either dye to the same extent. Air + O_2_ DBD plasma has strong action against the decolorization of both dyes, as compared to other gases DBD plasma. But as seen in the experimental section above, the discharge power differs depending upon the gas used in the plasma reactor. Therefore, in order to know which gas plays a strong role in the plasma reactor for the decolorization of dyes, we calculated the efficiency (g/kWh) for each dye, and for each gas.

**Figure 6:**
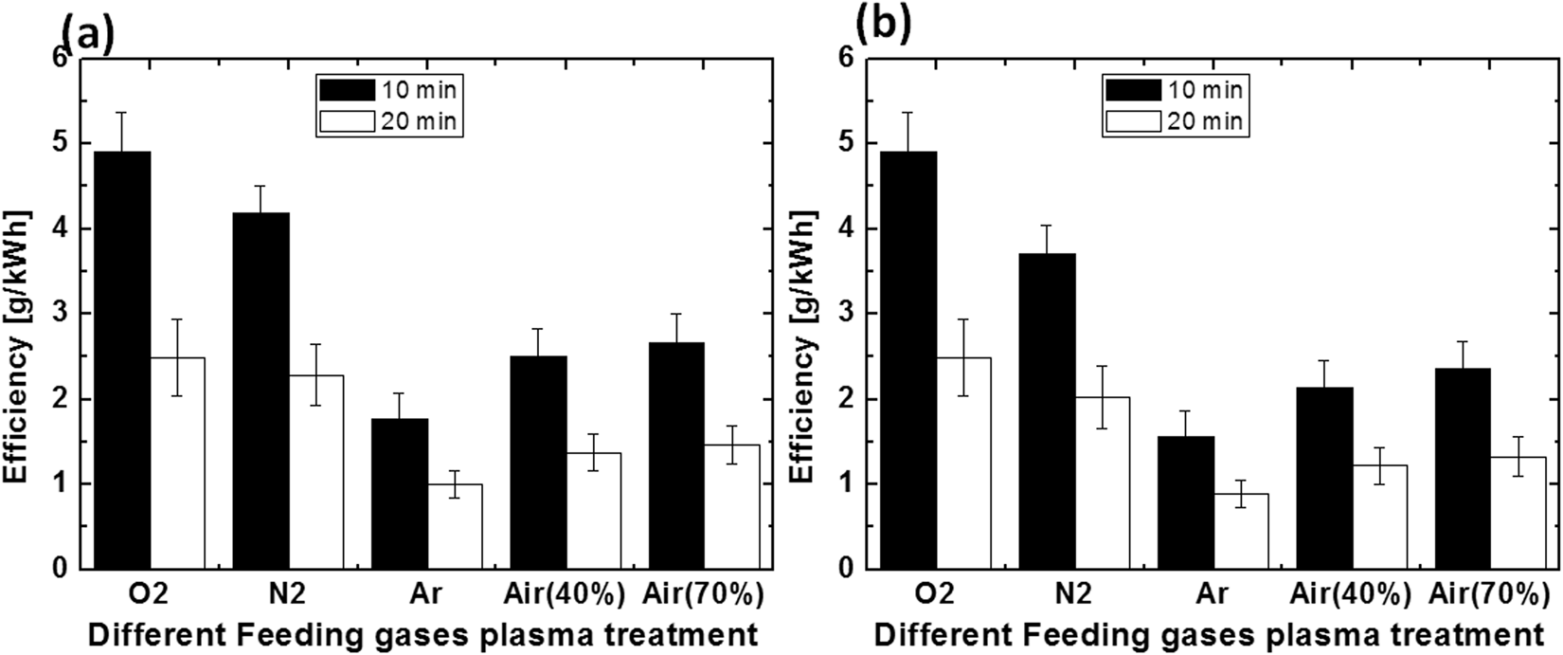
Energy efficiency of DBD plasma treatments for (10 and 20) min with (**a**) MO dye, and (**b**) MB dye.

Figure 6(a) shows that for DBD plasma treatment of the degradation of methyl orange in the presence of Air + O_2_, Air + N_2_, Air + Ar, and Air, respectively with humidity of 40%, the efficiency of 10 min treatment was (4.9, 4.1, 1.7, and 2.5) g/kWh; whereas, the efficiency of 20 min treatment was (2.4, 2.2, 1.0, and 1.3) g/kWh, respectively. Meanwhile, Fig. 6(b) shows that under the same conditions, for treatment of methylene blue, the efficiency of 10 min treatment was (4.9, 3.7, 1.5, and 2.1) g/kWh; while the efficiency of 20 min treatment was (2.4, 2.0, 0.8, and 1.2) g/kWh, respectively. Additionally, for treatment with Air DBD plasma, but for humidity of 70%, the efficiency for treatment of methyl orange for (10 and 20) min was (2.6 and 1.4) g/kWh, respectively; whereas, the efficiency for treatment of methylene blue was (2.3 and 1.3) g/kWh, respectively.

We have observed that for all the treated plasma conditions, the 10 min treatment showed higher efficiency than the 20 min treatment. This means that the rate of decolorization increases for 10 min for all plasma treatments (independent of the feeding gases), but later, the decolorization rate slows down, compared with the increase in treatment time. The work of Grabowski *et al*. suggested several possibilities for such a process^37^. The first possibility was that the uptake of ozone decreases with decrease in pH^37^. Hence, less O_3_ is available for the direct oxidation of dyes. The second possibility is that the decay product of the methyl orange and methylene blue forms a complex with the RS that may interfere with the absorption measurements. Thirdly, the dye degradation rate may reach saturation values after 10 min treatment, and further treatment doesn’t increase the dye degradation rate.

### Plasma simulation to understand the generation of reactive species in different feeding gases condition

**Figure 7:**
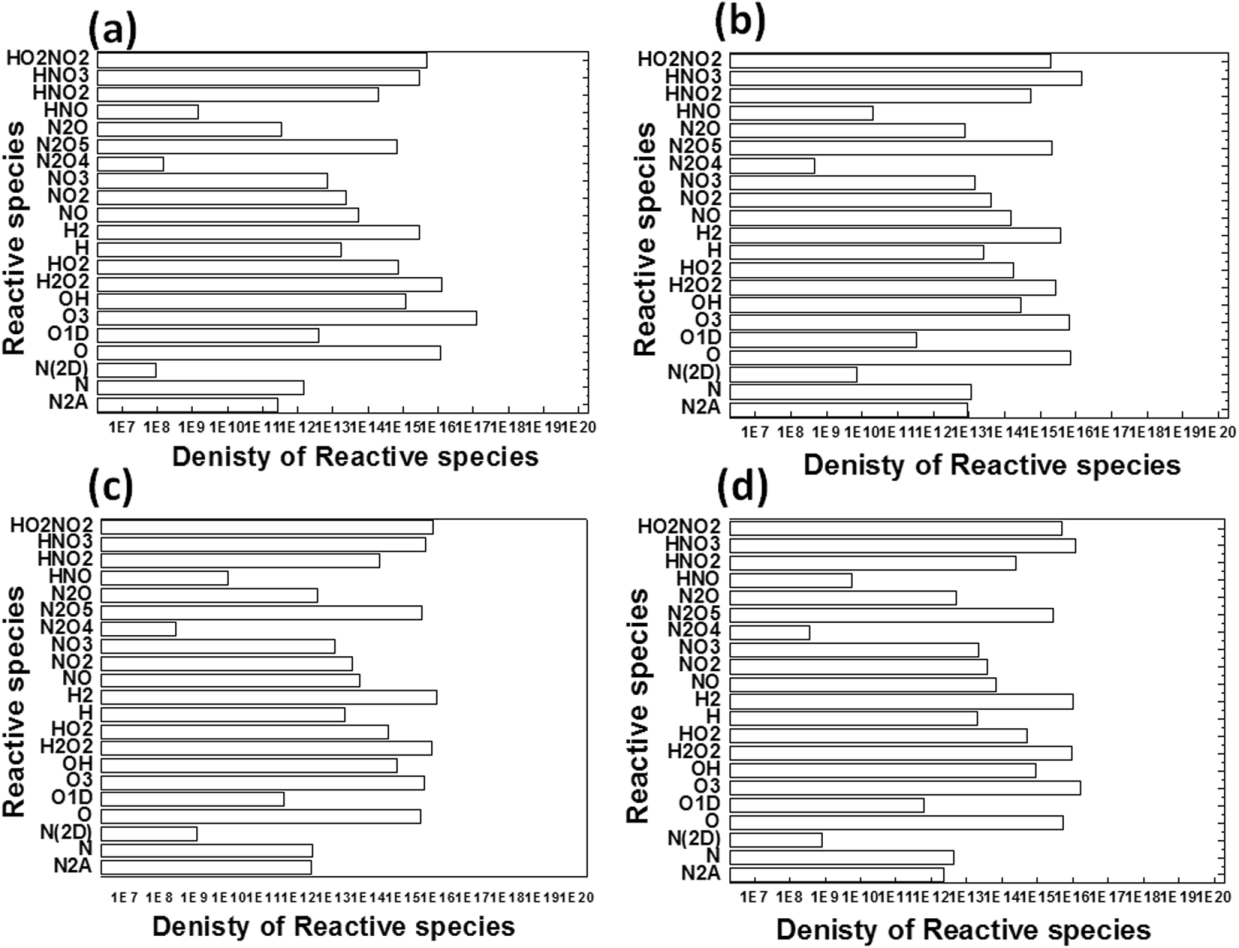
Simulation of reactive species generated using DBD plasma for different gases conditions (**a**) Air (40%) and O_2_ (60%); (**b**) Air (40%) and N_2_ (60%); (**c**) Air (40%) and Ar (60%); and (**d**) Air (100%); and having humidity of 40% in all cases.

**Figure 8:**
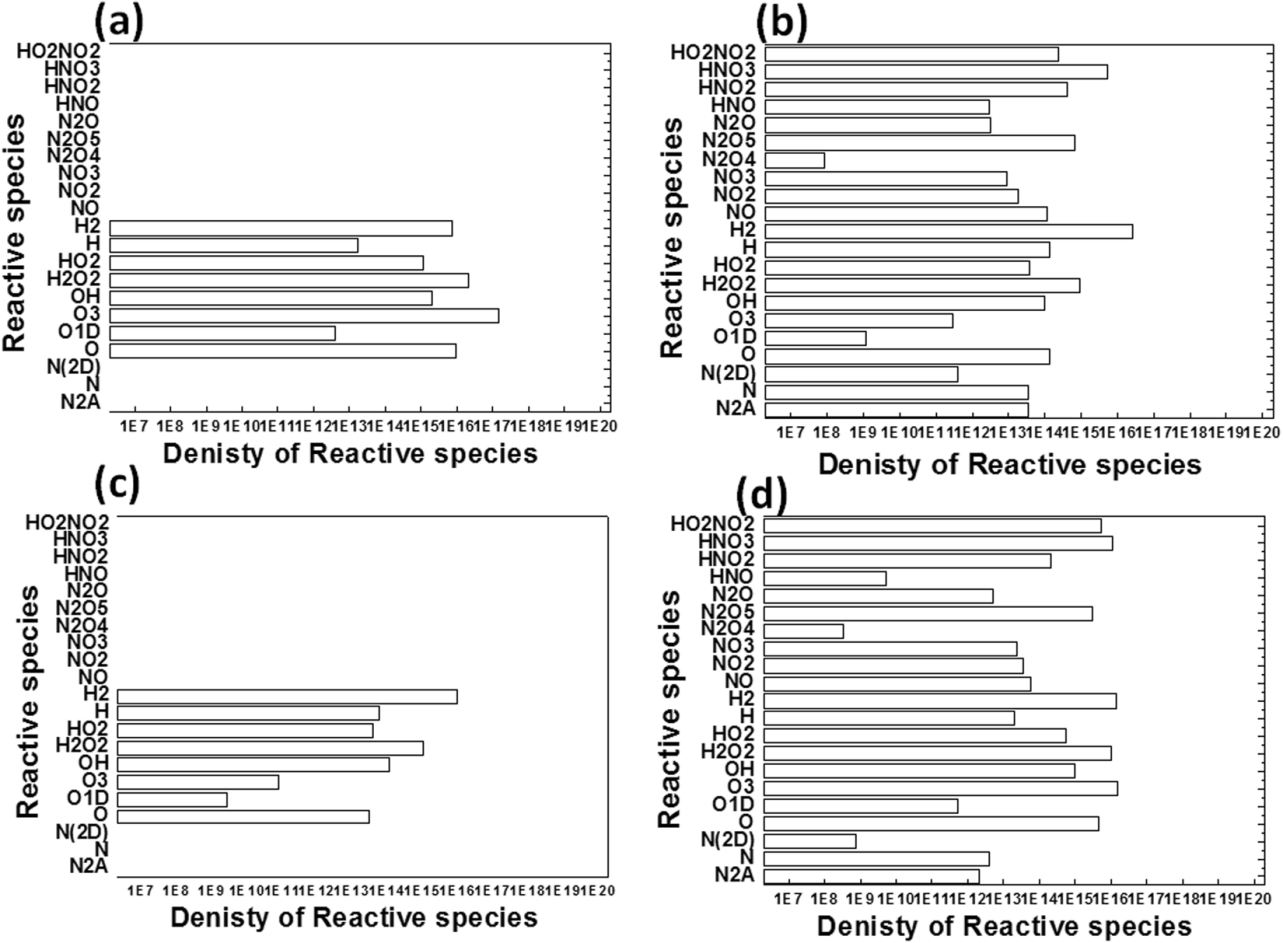
Simulation of reactive species generated using DBD plasma for different pure gases conditions (**a**) O_2_ with humidity of 40%; (**b**) N_2_ with humidity of 40%; (**c**) Ar with humidity of 40%; and (**d**) Air (100%) with humidity of 70%.

The above results show that RS generated from DBD plasma tend to decolorize the dyes (methyl orange and methylene blue). These RS have high reduction potential that increases the reaction between the dyes and RONS. The standard reduction potential of OH, O_3_, H_2_O_2_, HO_2_, O, ONOO^•^, O_2_NOO, NO_2_^•^, and N_2_O are given as (+2.8, +2.1, +1.77, +1.7, +2.42, +1.37, 1.59, 1.04, and 1.59) V^48–50^. This shows that OH, O, and O_3_ have high reduction potential, so they can react with the dyes, and result in degradation of the dyes. So to know the probable density of these highly RS generated in our studied DBD plasma system, we simulated the RS in two different conditions: (1) Fig. 7 shows that we used the DBD reactor with different feeding gases, such as ambient air and combination of Air + O_2_, +N_2_, and +Ar gases, with humidity of 40%; and (2) Fig. 8 shows probable density of reactive species generated in the presence of pure gases O_2_, N_2_, and Ar gases with humidity of 40%. During the simulation of RS, we assumed that the electron temperature was 1 eV, electron density was 5 × 10^15^ cm^−3^, and the gas residence time was 0.1 s. Using these assumptions, we predicted many reactions that are given in the supporting information. Some of the different RS generated in the presence of ambient air and combination of Air + O_2_, +N_2_, and +Ar gases, with humidity of 40%, are the following: N_2_A, N, N(2D), O, O1D, O_3_, OH, H_2_O_2_, HO_2_, H, H_2_, NO, NO_2_, NO_3_, N_2_O_4_, N_2_O_5_, N_2_O, HNO, HNO_2_, HNO_3_, and HO_2_NO_2_, in different conditions. The density of these species depends upon the feeding gases in the plasma reactor. In the first case with 60% O_2_, 40% Air, and 40% humidity, the density of RS with high oxidation potential, of O, O1D, O_3_, OH, H_2_O_2_, HO_2_, was (10^16^, 10^12^, 10^17^, 10^15^, 10^16^, and 10^14^)/cm^3^, respectively; similarly under a different simulation condition of 60% N_2_, 40% Air, and 40% humidity, the density of RS was (10^15^, 10^11^, 10^15^, 10^14^, 10^15^, and 10^14^)/cm^3^, respectively; for 60% Ar, 40% Air, and 40% humidity, the density of RS was (10^15^, 10^11^, 10^15^, 10^14^, 10^15^, and 10^14^)/cm^3^, respectively; and for pure ambient air with humidity of 40%, the density was (10^15^, 10^11^, 10^16^, 10^14^, 10^15^, and 10^14^)/cm^3^, respectively.

Figure 8 shows that in another simulation of gas conditions, we used pure O_2_, N_2_, and Ar gases with humidity of 40% having electron temperature 1 eV, electron density 5 × 10^15^ cm^−3^, and gas residence time of 0.1 s. The RS density in the presence of pure O_2_ with 40% humidity for O, O1D, O_3_, OH, H_2_O_2_, and HO_2_ was (10^15^, 10^12^, 10^17^, 10^15^, 10^16^, and 10^15^)/cm^3^, respectively; whereas, the RS density for pure N_2_ with 40% humidity, was (10^14^, 10^9^, 10^1^, 10^13^, 10^14^, and 10^13^)/cm^3^, respectively; for pure Ar plasma with 40% humidity, the RS density was (10^13^, 10^9^, 10^10^, 10^14^, 10^15^, and 10^13^)/cm^3^, respectively; and in the presence of pure Air with 70% humidity, the RS density was (10^15^, 10^11^, 10^16^, 10^14^, 10^15^, and 10^14^)/cm^3^. These simulation studies show that the densities of O, O_3_, and OH are high in given plasma conditions and due to their high reduction potential it helps in the degradation of dyes.

## Conclusion

In this study, we can conclude that plasma reactors with different feeding gases and gamma rays have strong potential to decolorize methyl orange and methylene blue dyes. The DBD plasma with O_2_ as feeding gas leads to more decolorization in less treatment time than any other feeding gas DBD plasma, which can be understood by the fact that more oxygen species can be transferred to the dye solution, which increases the decolorization efficiency. When comparing the energy efficiency of DBD plasma in all feeding gas systems, the O_2_ gas plasma shows better results for both dyes, precisely because it has low discharge power, and still gives considerable decolorization. Moreover, N_2_ gas plasma has the next highest energy efficiency after the O_2_ gas plasma, while the Ar gas plasma has the least energy efficiency, which is due to the large discharge power. Nevertheless the energy efficiency for Air plasma increases as the humidity increases from (40 to 70) %, but the difference between the two is not significant. Comparison of our present plasma system with the previous published work by different research groups on the degradation of methyl orange or methylene blue dye reveals that different research groups used different type of plasma reactors, but the efficiency (g/kWh) was not reported in many research articles, so it was hard to compare them^11,12,34–37^. Huang *et al*. used 35 min treatment with board-DBD plasma reactor to obtain 99% degradation of methyl orange^11^. The same author using DBD reactor for 40 min also reveals the degradation of methylene blue to ≈99% in the acidic solution, ≈91% in alkaline solution, and ≈75% in neutral solution treatment^12^. Another group using 15 min treatment with glow discharge plasma shows 93% decolorization of methyl orange solution^34^. Further, Chen *et al*. shows the degradation of methyl orange to ≈100% using Ar/O_2_ DBD plasma along with the Co_3_O_4_ NPs catalyst^36^. Recently, the same authors using 15 min treatment with byproducts of APPJ along with Ag/TiO_2_ nanocomposite, and having energy efficiency of about 0.69 g/kWh, show the degradation of methyl orange to 90%^35^. Grabowski *et al*. using 20 min treatment with pulsed corona discharge, having a yield of 4.5 g/kWh, shows the conversion of ≈90% of methylene blue and methyl orange^37^. However, our 10 min treatment of O_2_ DBD plasma, having a yield of 4.9 g/kWh, shows ≈98% degradation for both methylene blue and methyl orange, which shows that it was one of the better plasma systems for the decolorization of methylene blue and methyl orange. Additionally, the concentration limit of nitrite is 0.5 mg/l, and the nitrate concentration limit should not be more than 50 mg/l, according to the European drinking water regulations^51^. As shown above, O_2_ gas plasma system generates (4.4 and 6.4) μM of nitrite and nitrate concentrations, respectively, in the solution, which are lower than the permissible limits. Therefore, we believe that the DBD reactor with O_2_ feeding gas setup is appropriate for water purification.

To understand the mechanism of dye decolorization after the gamma rays and plasma treatments, we studied the various RONS concentrations in DI water. Among all the studied RONS, gamma rays show higher concentration of H_2_O_2_, as compared to NO_2_^−^ and NO_3_^−^. The concentration of RONS increases as the treatment time of gamma rays increases, and also decolorization increases as the treatment time increases. Although in the present study, the decolorization of dyes was not significant, it could increase with further increase in treatment time. This indicates that the concentrations of RONS generated during the gamma rays treatment in the present study, were not enough for the decolorization of dyes.

Whereas, DBD plasma treatment in different feeding gases resulted in high amounts of decolorization of the dyes. Therefore, if we compare the RS generated between the various gases plasma we can conclude that O_2_ gas plasma has higher amounts of H_2_O_2_, O_3_, and OH species, as compared to other feeding gases. Moreover, the decolorization of the methyl orange and methylene blue was also high for O_2_ gas plasma, showing almost 98% decolorization after 10 min treatment. As shown in our previous work^24^, H_2_O_2_ alone is unable to decolorize both dyes. Hence OH and O_3_ are the main species that are responsible for the decolorization of dyes, along with O (concentration not known). Comparison of the decolorization efficiency of O_2_ plasma and air plasma shows that after 10 min treatment, the methylene blue was decolorized to (~98 and ~72) % for air plasma. To understand this difference in the decolorization, we need to look at the change of pH of DI water after treatment with O_2_ plasma and air plasma after 10 min treatment. According to Grabowski *et al*.^37^, the pH plays an important role in methylene blue oxidation, because at low pH, the uptake of O_3_ decreases, and at higher pH, the more O_3_ diffuses to the liquid phase. This might be the reason that in our O_2_ plasma reactor, the decolorization is greater than for the air plasma, because in O_2_ plasma, the pH decreases to 5.1, and in air plasma, it decreases to 2.5. Additionally the high concentration of NO_2_^−^ for air plasma also decreases the O_3_ concentration in solution. Hence, less concentration of O_3_ is available for the reaction with dyes, therefore the efficiency of the air plasma is low, as compared to the O_2_ plasma. Moreover, as per the literature^52–54^, O_3_ attacks the molecule in two ways, firstly, direct oxidation of the target molecule by O_3_, and secondly, O_3_ is converted to OH radicals, and then oxidizes the target molecules. In our previous work, we have shown the possible reactions between the OH and methylene blue and methyl orange using DFTB-MD simulations^24^. This shows that in O_2_ plasma, both O_3_ and OH are predominant radicals for the reactions with dyes. Whereas, for the air plasma, due to decrease in the pH, the uptake of O_3_ decreases, hence the OH is main radical used for the degradation of dyes. That is why even though O_3_ is high for air plasma compared to the N_2_ and Ar plasma, the decolorization efficiency is quite close to theirs. On the other hand, N_2_ and Ar plasma has low O_3_ concentration, but having high OH radical concentration as compared to air plasma. While for O_2_ plasma, both O_3_ and OH are quite high, hence this treatment has the maximum decolorization effect. The simulation studies also show that O_2_ plasma has high densities of O_3_ and OH, of (10^17^ and 10^15^)/cm^3^, respectively, which is more than for the other conditions. Hence, the high densities of O_3_ and OH play an important role in the decolorization of dyes, due to their high reduction potential. Therefore, the DBD plasma reactor with O_2_ feeding gas provides high decolorization efficiency, along with high energy efficiency. Hence, this study supports that DBD plasma with O_2_ feeding gas can play a potential role in wastewater purification.

## Electronic supplementary material

Supporting Information
