# Supplementary material for: Impact of Gamma rays and DBD plasma treatments on wastewater treatment
Source: Sci Rep. 2018 Feb 13;8:2926. doi: 10.1038/s41598-018-21001-z (PMC5811431; doi:10.1038/s41598-018-21001-z)
Supplement: Supplementary file 1 — Supporting Information [file 41598_2018_21001_MOESM1_ESM.pdf]

Supporting information

Impact of Gamma rays and DBD plasma treatments on wastewater treatment

Pankaj Attri[1,2], Fumiyoshi Tochikubo[3], Ji Hoon Park[1], Eun Ha Choi[1], Kazunori Koga[2], and Masaharu Shiratani[2]

[1]Plasma Bioscience Research Center/Department of Electrical and Biological Physics, Kwangwoon University, Seoul 01897, Korea.
[2]Faculty of Information Science and Electrical Engineering, Kyushu University, Fukuoka, Japan.
[3]Department of Electrical and Electronic Engineering, Tokyo Metropolitan University, Tokyo

Probable reactions with their corresponding rate coefficient used in simulations for Air and Air + O_2, + N_2 and + Ar.

											Rate coefficient [cm3s-1]	Reference
e	+	N2			-->	N2(a')	+	e			6.05E-15	Boltz
e	+	N2			-->	N2(A)	+	e			2.08E-14	Boltz
e	+	N2			-->	N	+	N	+	e	1.30E-16	Boltz
e	+	N			-->	N(2D)	+	e			1.34E-10	Boltz
e	+	N2(A)			-->	N2	+	2e			1.91E-14	Boltz
e	+	O2			-->	O2(1delta)	+	e			4.75E-11	Boltz
e	+	O2			-->	O	+	O	+	e	6.36E-13	Boltz
e	+	O2			-->	O(1D)	+	O	+	e	1.35E-13	Boltz
e	+	O			-->	O(1D)	+	e			1.15E-10	Boltz
e	+	H2O			-->	O	+	H2			2.14E-18	Boltz
e	+	H2O			-->	H	+	OH	+	e	2.80E-14	Boltz
e	+	Ar			-->	Ar*	+	e			3.95E-17	Boltz
e	+	O3			-->	e	+	O2	+	O(1D)	5.0e-10*Te^-0.5	1
O	+	O2	+	M	-->	O3	+	M			6.9E-34*(T/300)^-1.25	4

H	+	OH	+	M	-->	H2O	+	M			4.30E-31	2
N2O5					-->	NO2	+	NO3			5.49e14*T^0.1*exp(-11080/T)	4
NO	+	O2	+	NO	-->	NO2	+	NO2			1.40E-38	4
NO	+	HO2			-->	NO2	+	OH			3.7e-12*exp(240/T)	5
N(2D)	+	N2			-->	N	+	N2			2.40E-14	2
NO	+	O	+	M	-->	NO2	+	M			2.44e-27*T^-1.8	5
N(2D)	+	O2			-->	NO	+	O			6.80E-12	2
NO	+	OH	+	M	-->	HNO2	+	M			7.41e-31*(T/300)^-2.4	5
N(2D)	+	NO			-->	N2	+	O			6.30E-11	2
NO	+	H	+	M	-->	HNO	+	M			3.40E-32	2
N(2D)	+	N2O			-->	NO	+	N2			2.60E-12	2
NO	+	NO3			-->	NO2	+	NO2			1.6e-11*exp(150/T)	5
HO2	+	NO	+	M	-->	HNO3	+	M			5.60E-33	4
HO2	+	NO			-->	O2	+	HNO			9.0e-19*exp(2819/T)	4
N(2D)	+	NO2			-->	N2O	+	O			1.50E-12	2
NO	+	O3			-->	NO2	+	O2			2.0e-12*exp(-1400/T)	4
N(2D)	+	NO2			-->	NO	+	NO			1.50E-12	2
NO	+	N			-->	N2	+	O			3.10E-11	2
H	+	O2	+	M	-->	HO2	+	M			5.64e-28*T^-1.6	2
H	+	H	+	M	-->	H2	+	M			4.80E-33	2
H	+	NO2			-->	OH	+	NO			8.00E-11	2
H	+	HO2			-->	OH	+	OH			2.8e-10*exp(-440/T)	4
N2(A)	+	N2			-->	N2	+	N2			3e-16*(T/300)^0.5	6
H	+	O3			-->	OH	+	O2			1.4e-10*exp(-480/T)	5
N2(A)	+	NO			-->	N2	+	NO			6.90E-11	6
H	+	HNO			-->	H2	+	NO			1.00E-11	2
N2(A)	+	O2			-->	N2	+	O	+	O	1.70E-12	10
N	+	NO2			-->	N2O	+	O			2.40E-12	2
N2(A)	+	O2			-->	N2	+	O2(1delta)			7.50E-13	

N	+	NO2			-->	NO	+	NO			6.00E-13	2
N2(A)	+	N2O			-->	N2	+	N2	+	O	1.40E-11	10
N2(A)	+	N2O			-->	N2	+	N2O			1.70E-10	10
N	+	N	+	M	-->	N2	+	M			3.9e-33*(T/300)^-1.5	2
N2(A)	+	NO2			-->	N2	+	NO	+	O	1.30E-11	6
N2(A)	+	H2			-->	N2	+	H2			2.60E-11	10
N	+	OH			-->	NO	+	H			3.8e-11*exp(85/T)	5
O(1D)	+	N2			-->	O	+	N2			1.8e-11*exp(107/T)	5
N	+	O	+	M	-->	NO	+	M			5.46e-33*exp(155/T)	11
O(1D)	+	O2			-->	O	+	O2			3.80E-11	2
N	+	O2			-->	NO	+	O			4.4e-12*exp(-3220/T)	5
N	+	O3			-->	NO	+	O2			5.00E-16	2
O(1D)	+	H2O			-->	O	+	H2O			1.20E-11	2
O(1D)	+	H2O			-->	OH	+	OH			2.20E-10	2
O	+	HO2			-->	OH	+	O2			2.9e-11*exp(200/T)	5
O	+	O3			-->	O2	+	O2			8.0e-12*exp(-2060/T)	5
O	+	NO2	+	M	-->	NO3	+	M			9.0e-32*(T/300)^-2.0	5
NO3	+	NO3			-->	NO2	+	NO2	+	O2	1.20E-15	2
OH	+	OH			-->	O	+	H2O			3.5e-16*T^1.4*exp(200/T)	7
N2O5	+	H2O			-->	HNO3	+	HNO3			5.00E-21	2
OH	+	NO2	+	M	-->	HNO3	+	M			2.2e-30*(T/300)^-2.9	5
OH	+	HNO3			-->	NO3	+	H2O			1.5e-14*exp(650/T)	2
OH	+	HNO			-->	H2O	+	NO			2.15e-17*T^1.88*exp(481/T)	4
OH	+	HO2			-->	H2O	+	O2			8.00E-11	1
OH	+	HNO2			-->	NO2	+	H2O			1.8e-11*exp(-390/T)	5
OH	+	O3			-->	HO2	+	O2			1.9e-12*exp(-1000/T)	2
OH	+	N2O			-->	HNO	+	NO			3.80E-17	2
HO2	+	NO2	+	M	-->	HO2NO2	+	M			1.5e-31*(T/300)^-3.2	5
HO2	+	O3			-->	OH	+	O2	+	O2	1.4e-14*exp(-600/T)	2

NO2	+	O3			-->	NO3	+	O2			1.2e-13*exp(-2450/T)	5
O	+	NO2			-->	NO	+	O2			6.5e-12*exp(120/T)	5
O	+	NO3			-->	NO2	+	O2			1.00E-11	2
O	+	OH			-->	H	+	O2			2.3e-11*exp(110/T)	7
OH	+	OH	+	M	-->	H2O2	+	M			6.9e-31*(T/300)^-0.8	5
OH	+	H2O2			-->	H2O	+	HO2			2.9e-12*exp(-160/T)	2
H	+	HO2			-->	H2	+	O2			1.1e-10*exp(-1070/T)	7
HO2NO2	+	M			-->	HO2	+	NO2	+	M	3.6e-6*exp(-10000/T)	2
HO2NO2	+	M			-->	HO2	+	NO2	+	M	5e-6*exp(-10000/T)	5
OH	+	H2			-->	H2O	+	H			7.7e-12*exp(-2100/T)	7
O(1D)	+	H2O			-->	H2	+	O2			2.30E-12	2
O(1D)	+	H2			-->	OH	+	H			1.10E-10	2
O	+	H2O2			-->	OH	+	HO2			1.4e-12*exp(-2000/T)	7
O	+	H2			-->	OH	+	H			1.6e-11*exp(-4570/T)	2
H	+	HO2			-->	H2O	+	O			9.40E-13	2
HO2	+	HO2	+	M	-->	H2O2	+	O2	+	M	1.9e-33*exp(980/T)	5
NO2	+	NO3	+	M	-->	N2O5	+	M			2.7e-30*(T/300)^-3.4	5
O	+	H	+	M	-->	OH	+	M			1.62E-32	12
H	+	H2O2			-->	H2O	+	OH			4e-11*exp(-2000/T)	7
NO2	+	NO2	+	M	-->	N2O4	+	M			1.4e-33*(T/300)^-3.8	13
N2O4	+	M			-->	NO2	+	NO2	+	M	1.29e-5*(T/300)^-3.8*exp(-6460/T)	13
OH	+	NO3			-->	HO2	+	NO2			2.60E-11	15
HO2	+	NO3			-->	OH	+	NO2	+	O2	3.60E-12	15
HO2	+	NO3			-->	HNO3	+	O2			9.20E-13	15
H	+	H2O2			-->	HO2	+	H2			8e-11*exp(-4000/T)	7
HNO3	+	NO			-->	HNO2	+	NO2			7.37E-21	16
H2	+	O2			-->	H	+	HO2			2.4e-10*exp(-28500/T)	7
H	+	O2			-->	OH	+	O			2.8e-7*T^-0.9*exp(-8759/T)	7
OH	+	M			-->	O	+	H	+	M	4e-9*exp(-50000/T)	7

OH	+	O2			-->	O	+	HO2			3.7e-11*exp(-26500/T)	7
OH	+	H			-->	O	+	H2			1.14e-12*T^0.67*exp(-518/T)	3
HO2	+	M			-->	H	+	O2			2e-5*T^-1.18*exp(-24363/T)	7
HO2	+	H2			-->	H2O2	+	H			5e-11*exp(-13100/T)	7
H2O2	+	O2			-->	HO2	+	HO2			9e-11*exp(-20000/T)	7
H	+	H2O			-->	H2	+	OH			1.03e-16*T^1.9*exp(-9265/T)	7
O	+	H2O			-->	OH	+	OH			7.6e-15*T^1.3*exp(-8605/T)	7
O	+	OH	+	M	-->	HO2	+	M			2.76E-31	4
O(1D)	+	O3			-->	O2	+	O	+	O	1.20E-10	4
O(1D)	+	NO			-->	O2	+	N			8.50E-11	4
OH	+	NO2			-->	HO2	+	NO			3.03e-11*exp(-3360/T)	4
NO2	+	NO3			-->	NO	+	NO2	+	O2	8.21e-14*exp(-1480/T)	4
O(1D)	+	NO2			-->	O2	+	NO			2.50E-10	4
O(1D)	+	N2O			-->	NO	+	NO			6.70E-11	4
O3	+	NO2			-->	O2	+	O2	+	NO	1.00E-18	4
O	+	HNO			-->	OH	+	NO			1.82E-11	4
HNO	+	O2			-->	NO	+	HO2			5.25e-12*exp(-1510/T)	4
O	+	O	+	M	-->	O2	+	M			5.21e-35*exp(900/T)	4
HO2	+	NO2			-->	HNO2	+	O2			1.20E-13	17
O	+	HNO2			-->	NO	+	OH			2e-11*exp(-3000/T)	17
N2(a')	+	N2			-->	N2(A)	+	N2			2.00E-13	18
N2(a')	+	O2			-->	N2	+	O	+	O	2.80E-11	18
N2(a')	+	NO			-->	N2	+	N	+	O	3.30E-11	19
N2(A)	+	H2O			-->	N2	+	OH	+	H	5.00E-14	6
N2(a')	+	H2O			-->	N2	+	H2O			3.00E-10	19
Ar*	+	H2O			-->	Ar	+	OH	+	H	2.10E-10	21
Ar*	+	O2			-->	Ar	+	O	+	O*	2.10E-10	22
Ar*	+	O			-->	Ar	+	O*			4.10E-11	23

References

1 R. Dorai, Ph.D Thiesis, Univ. Illinois at Urban-Champaign, 2002.
2 J.C.Person and D.O. Ham, Radiat. Phys. Chem., Vol. 31, 1 (1988)
3 B. R. Rowe, F. Valle, J. L. Queffelec, J. C. Gomet and M. Morlais, J. Phys. Chem. Vol. 88, 845 (1988)
4 Y. Morikin and W.G. Mallard, The NIST Chemical Kinetics Database-Version 2Q98 (1998)
5 R. Atkinson, D.K. Baulch, R.A. Cox, R.F. Hampson, Jr., J.A. Kerr and J. Tore, J. Phys. Chem. Ref. Data Vol. 18, 881 (1989)
6 J.T. Herron, J. Phys. Chem. Ref. Data, Vol.28, 1453 (1999)
7 W.Tsang and R.F. Hampson, J. Phys. Chem. Ref. Data Vol. 21, 1125 (1992)
8 S. Mullavilli, C.K. Lee, K. Varghese and L.L. Tavlarides, IEEE Trans. Plasma Sci. Vol.16, 652 (1988)
9 R.E. Olson, J.R. Petersonand J. Moseley, J. Chem. Phys. Vol.53, 3391 (1970)
10 L.G. Piper, J. Chem. Phys. Vol. 87, 1625 (1987)
11 R. Atkinson et al., J. Phys. Chem. Ref. Data, Vol. 15, 1087 (1992)
12 R.K. Bera and R. J. Hanrahan, J. Appl. Phys., Vol. 62, 2523 (1987)
13 P. Borrell, C. J. Cobos and K. Luther, J. Phys. Chem. Vol. 92, 4377 (1988)
14 W. Hack, P. Rouveirolles and H.G. Wagner, J. Phys. Chem., Vol.90, 2505 (1986)
15 A. Mellouki, G. Le Bras and G. Poulet, J. Phys. Chem., Vol. 92, 2229 (1988)
16 R. Svensson and E. Ljungstrom, Int. J. Chem. Kin., Vol. 20, 857 (1988)
17 W.G. Mallard, F. Westley, J.T. Herron and R.F. Hampson, NIST Chemical Kinetics Database-Ve rsion 6.0, (1994)
18 I.A. Kossyi, A. Yu Kostinsky, A.A. Matveyev and V.P.Silakov, Plasma Sources Sci. Technol., Vo l. 1, 207 (1992)
19 F. Fresnet, G. Baravian, L. Magne, S. Pasquiers, C. Postel. V. Puech and A. Rousseau, Plas ma Sources Sci. Technol., Vol. 11, 152 (2002)
20 D.X. Liu, P. Bruggeman, F. Iza, M.Z. Rong and M.G. Kong, Plasma Souces Sci. Technol., Vol. 19, 025018 (2010)
21 T. Shirafuji and T. Murakami, Jpn. J. Appl. Phys., Vol. 54, 01AC03 (2015)
22 L.G. Piper, J. E. Velazco and D.W. Sester, J. Chem. Phys., Vol. 59, 3323 (1973)
23 D.L. King, L. G. Piper and D.W. Sester, J. Chem. Soc. Faraday Trans., Vol 73, 177 (1977)

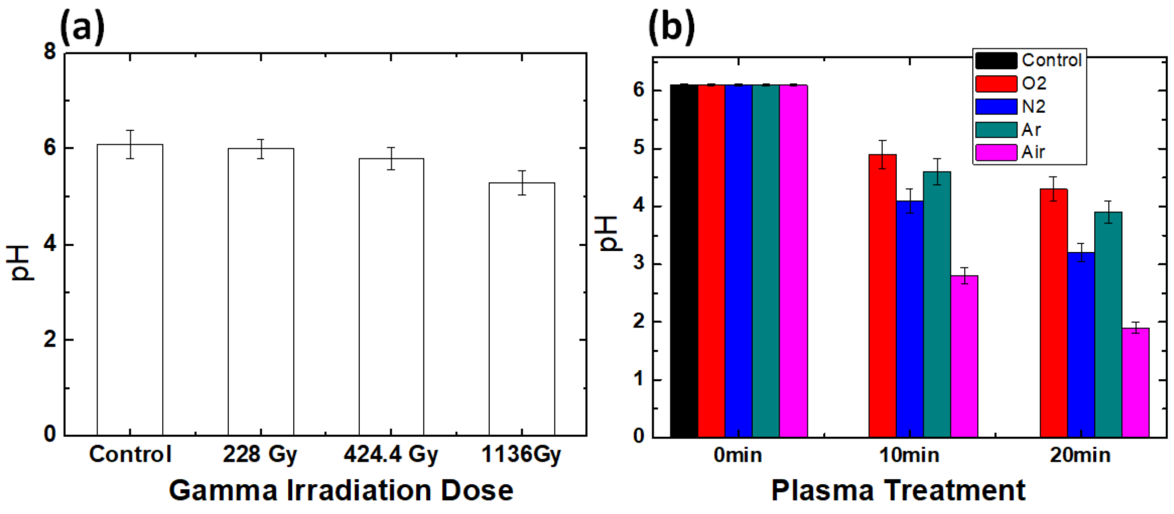

Figure S1: Change in pH of water (a) after Gamma treatment and (b) after plasma treatment
